# Four Acid-Base Disturbances in a Critically-Ill Patient Undergoing Emergent Abdominal Surgery

**DOI:** 10.1155/2022/1285598

**Published:** 2022-07-04

**Authors:** Orestes Mavrothalassitis, Balkarn S. Thind, Ashish Agrawal

**Affiliations:** Department of Anesthesia and Perioperative Care, University of California San Francisco, San Francisco, CA, USA

## Abstract

Lactic acidosis is common in critically-ill surgical patients, but not all perioperative acid-base imbalances are attributable to tissue hypoperfusion. Other causes of acid-base abnormalities can be missed when focused on acute resuscitation of a surgical pathology. This report presents the case of a 60-year-old woman with no past medical history who underwent exploratory laparotomy for umbilical hernia with incarcerated and perforated bowel whose perioperative management was complicated by four acid-base disturbances, including starvation ketosis. This case highlights the importance of early recognition of acid-base imbalances to explain concurrent medical pathology and accurately predict a patient's expected post-operative course.

## 1. Introduction

Acid-base disturbances are frequent among critically-ill patients undergoing surgery. The most common abnormality is lactic acidosis due to tissue hypoperfusion or anaerobic glycolysis, generally treated with resuscitation, antibiotics, or urgent surgery depending on the initial cause. However, not all perioperative metabolic acidosis is from tissue hypoperfusion, and there can be simultaneous processes occurring in the same patient. In this report we describe the case of a woman with no past medical history who presented with bowel perforation and whose perioperative course was complicated by four separate acid-base abnormalities. The patient provided written Health Insurance Portability and Accountability Act (HIPPA) authorization to publish this case report.

## 2. Case Presentation

**Figure 1:**
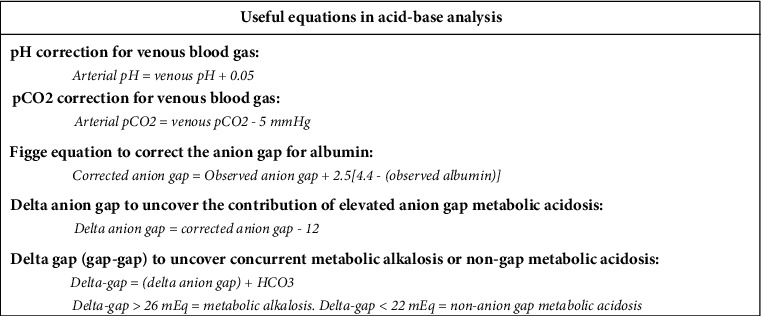
Useful equations for the evaluation of metabolic acidosis.

A 60-year-old woman with no past medical history and no prior surgeries presented to the emergency department with four days of abdominal pain and vomiting and two weeks of generalized weakness, non-bloody non-bilious emesis, and periumbilical abdominal pain with minimal appetite. Vitals on admission were heart rate 116, blood pressure 96/59, respiratory rate 20 breaths per minute, oxygen saturation 97% on room air, temperature 36.6 Celsius. Physical examination revealed a soft, non-distended but tender abdomen with erythema around the umbilicus with a yellow center draining serous fluid. Initial labs were notable for sodium 132, chloride 91, blood urea nitrogen 55 mg/dL, glucose 156 mg/dL, creatinine 0.88 mg/dL, venous pH 7.46, pCO2 30 mmHg, bicarbonate 21 mEq/L, lactate 3.3 mg/dL, base deficit 2.5 mEq/L, anion gap 19, and albumin 3.4 g/dL. Arterial correction for venous blood gas values demonstrated pH 7.51 and pCO2 25 mmHg (Figure 1). Therefore, the first two acid-base disturbances identified were an uncompensated primary respiratory alkalosis in addition to a metabolic lactic acidosis. The observed anion gap of 19 was corrected for albumin using the Figge equation yielding an adjusted anion gap of 21.5. Subtracting an expected anion gap of 12 from an observed anion gap of 21.5 yielded a delta anion gap of 9.5. Subtracting the lactate of 3.3 mg/dL left another 6.2 of unaccounted acid, indicating a third acid-base disturbance, an unexplained non-lactate elevated anion gap metabolic acidosis. A gap-gap was calculated to be 30.5 mEq/L, indicating the fourth acid-base disturbance to be a concomitant metabolic alkalosis from repeated emesis prior to presentation with gastric loss of hydrochloric acid.

Computed tomography scan of the abdomen demonstrated an umbilical hernia with incarcerated small bowel and free air consistent with a perforated viscus. Two liters of normal saline and ertapenem 1 gram were administered in the emergency department and the patient underwent exploratory laparotomy. Operative course was notable for washout of pus and feculent material and excision of 60 centimeters of necrosed small bowel. Estimated blood loss was 300 mL, and 1.5 L of plasmalyte and 1 L albumin were administered intraoperatively. Abdominal fascia was left open with a plan for subsequent re-exploration and the patient was transported intubated to the intensive care unit on norepinephrine (0.25 mcg/kg/min) and propofol (40 mcg/kg/min) infusions.

Postoperative course was notable for ongoing vasopressor requirement and worsening metabolic acidosis refractory to fluid resuscitation. An ABG drawn on postoperative day 1 showed a pH 7.28, pCO2 33 mmHg, bicarbonate 15.5 mEq/L, lactate 1.6 mg/dL, base deficit 11.2, anion gap 18, albumin 2.8 g/dL. Repeat exploratory laparotomy and washout did not demonstrate any new perforation, feculent material, or necrosis. Labs sent to further evaluate the patient's metabolic acidosis showed creatinine kinase 29 units/L (reference range 26-140 units/L), serum osmolality 298 mOsm/kg (reference range 280-301 mOsm/kg), and beta-hydroxybutyrate elevated to 5.8 mmol/L (reference range<0.28 mmol/L).

Given normal blood sugars, no history of diabetes, and pre-hospital anorexia, starvation ketosis was suspected. A 5% dextrose infusion was initiated at 50 cc/hr upon return to the intensive care unit, and the metabolic acidosis resolved within 12 hours. The patient underwent ileocecectomy, end ileostomy and abdominal wall closure on postoperative day three after initial presentation and was extubated in the intensive care unit post-operatively. After discontinuation of vasopressors and mechanical ventilation, venous acid-base analysis had normalized to pH 7.37, pCO2 47, base excess 1.9, bicarbonate 23, anion gap 7.

## 3. Discussion

**Figure 2:**
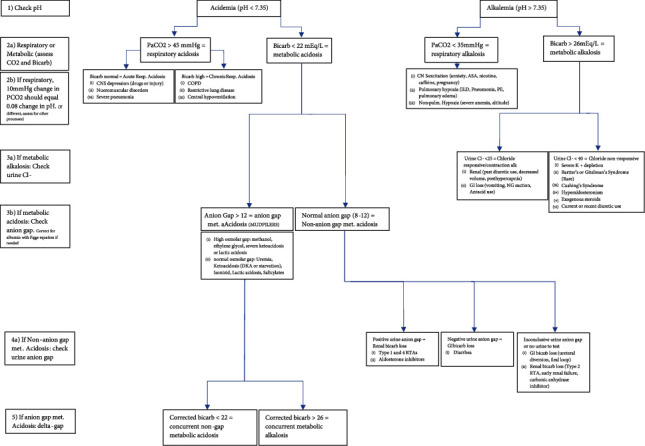
Comprehensive framework for acid-base interpretation.

Our case report of a critically-ill perioperative patient illustrates the importance of an organized and comprehensive approach to acid-base interpretation. Acid-base analysis is typically first approached by identification of the primary acid-base disturbance. Normal arterial pH is 7.40, with arterial pH less than 7.35 defining acidemia and a primary acidosis, and arterial pH greater than 7.45 defining alkalemia and a primary alkalosis. An acidosis is further characterized as metabolic (bicarbonate <22 mEq/L) or respiratory (pCO2>45 mmHg) and an alkalosis is likewise further characterized as metabolic (bicarbonate >26 mEq/L) or respiratory (pCO2<35 mmHg) [1]. The initial blood gas retrieved in the emergent setting is often venous, for which correction is appropriate (arterial blood gas [ABG] pH = venous blood gas [VBG] pH+0.05 and ABG CO2 = VBG pCO2 – 5 mmHg, Figure 1), though in critically-ill patients the corrections can be more variable [2, 3]. Our patient presented with arterially corrected initial blood gas values of pH 7.51 and pCO2 25 mmHg, indicating the presence of a primary respiratory alkalosis, most likely due to hyperventilation in response to abdominal pain and anxiety. However, the framework of a “primary disorder” implies a single etiology accompanied by physiologic compensation, whereas critically-ill patients can often have simultaneous processes and deserve further interpretation. A framework for comprehensive acid-base interpretation is outlined in Figure 2.

The next step to uncover any additional anion gap metabolic acidoses is to calculate a delta anion gap. To do this, an observed anion gap is first corrected for albumin using the Figge equation: Anion gap +2.5[(normal albumin) – (observed albumin)] [4]. In the case of our patient, this yielded 21.5 [(Observed anion gap 19+2.5(Normal albumin 4.4 g/dL - observed albumin 3.4 g/dL) =21 [5]. .A normal anion gap of 12 is subtracted from the albumin-corrected anion gap to calculate the delta anion gap. The clinician can next compare the calculated delta anion gap to known sources of anion gap metabolic acidosis and see if there are any unexplained contributors. In the case of our patient, the delta anion gap was 9.5 with the only known contributor being a lactate of 3.3 mg/dL, indicating the presence of an unexplained non-lactate elevated anion gap acidosis.

Once an anion gap acidosis has been evaluated, a delta gap or gap-gap calculation can uncover a concurrent non-gap metabolic acidosis or metabolic alkalosis. A gap-gap is calculated by adding the calculated delta anion gap to the measured serum bicarbonate [5–7]. Should the corrected bicarbonate deviate from normal, <22 or>26 mEq/L, there is a concurrent non-gap metabolic acidosis or metabolic alkalosis, respectively. In our patient, the delta anion gap of 9.5 added back to the bicarbonate was 30.5 mEq/L, suggesting a concomitant metabolic alkalosis explained by her repeated emesis and gastric loss of hydrochloric acid. Thus, on presentation this patient had four simultaneous processes: a respiratory alkalosis, a lactic acidosis, a starvation ketoacidosis, and a metabolic alkalosis.

Ketoacidosis is often not considered early in the workup and management of perioperative metabolic acidosis, especially if patients do not have a history of diabetes or present to the hospital hyperglycemic. However, some amount of ketosis has previously been demonstrated in 5% of patients undergoing surgery under general anesthesia [8]. Although ketosis can be minimal and transient after a single elective surgery, the condition can become profound after several days of fasting. Our patient experienced sustained anorexia prior to coming to the hospital with abdominal pathology and remained intubated without nutrition postoperatively, a common presentation.

Acid-base physiology can be complex, and clinicians are often confused by the strengths and weaknesses of any individual approach to a patient's acid-base status [9–11]. Furthermore, in vivo analyses can vary from theoretical in vitro experiments causing additional variability of “normal” ranges [12]. Although this patient ultimately had a favorable outcome, uncorrected acidosis can decrease the efficacy of vasopressors resulting in worsened tissue hypoperfusion. Acidosis will also increase the ventilatory effort necessary for respiratory compensation, which can lead to prolonged mechanical ventilation or failure of extubation and requirement for reintubation. Early identification and correction of acid-base disturbances is necessary to reduce the likelihood of organ dysfunction and mortality in critically-ill patients.

## 4. Conclusion

Critically-ill patients can often have multiple primary acid-base disorders. Having a systematic algorithm to interpret acid-base disturbances and keeping a wide differential for additional or concurrent pathologies can help the clinical team elucidate the underlying conditions, apply the correct therapies, and hasten the patient's recovery.

## Data Availability

As this is a case report, there is no underlying data except for the patient medical record which is protected.
